# Identification of Cellular Infiltrates during Early Stages of Brain Inflammation with Magnetic Resonance Microscopy

**DOI:** 10.1371/journal.pone.0032796

**Published:** 2012-03-12

**Authors:** Helmar Waiczies, Jason M. Millward, Stefano Lepore, Carmen Infante-Duarte, Andreas Pohlmann, Thoralf Niendorf, Sonia Waiczies

**Affiliations:** 1 Experimental and Clinical Research Center, a joint cooperation between the Charité Medical Faculty and the Max-Delbrück Center for Molecular Medicine, Berlin, Germany; 2 Berlin Ultrahigh Field Facility, Max-Delbrück Center for Molecular Medicine, Berlin, Germany; 3 Experimental Neuroimmunology, Charité Universitätsmedizin Berlin, Berlin, Germany; 4 Department of Anatomy, University of Malta, Msida, Malta; Institute Biomedical Research August Pi Sunyer (IDIBAPS) - Hospital Clinic of Barcelona, Spain

## Abstract

A comprehensive view of brain inflammation during the pathogenesis of autoimmune encephalomyelitis can be achieved with the aid of high resolution non-invasive imaging techniques such as microscopic magnetic resonance imaging (μMRI). In this study we demonstrate the benefits of cryogenically-cooled RF coils to produce μMRI *in vivo*, with sufficient detail to reveal brain pathology in the experimental autoimmune encephalomyelitis (EAE) model. We could visualize inflammatory infiltrates in detail within various regions of the brain, already at an early phase of EAE. Importantly, this pathology could be seen clearly even without the use of contrast agents, and showed excellent correspondence with conventional histology. The cryogenically-cooled coil enabled the acquisition of high resolution images within short scan times: an important practical consideration in conducting animal experiments. The detail of the cellular infiltrates visualized by *in vivo* μMRI allows the opportunity to follow neuroinflammatory processes even during the early stages of disease progression. Thus μMRI will not only complement conventional histological examination but will also enable longitudinal studies on the kinetics and dynamics of immune cell infiltration.

## Introduction

Inflammatory diseases of the central nervous system (CNS) such as multiple sclerosis (MS) involve a recruitment of immune cells during the early stages of pathogenesis, prior to the onset of clinical symptoms [1]–[3]. Normally the blood-brain barrier (BBB) restricts migration of immune cells to the CNS, but during inflammation its function becomes altered. Immune cells gain access to CNS parenchyma via a complex, multi-step process that involves crossing both the vascular endothelium and the glia limitans [4], [5]. The indirect detection of contrast-enhancing lesions (CEL) by Magnetic Resonance Imaging (MRI) at the site of BBB disruption as a result of contrast agent leakage into the CNS parenchyma is used as a primary end point in MS clinical trials [6], [7] and in the EAE mouse model [8], [9]. However, BBB disruption does not provide direct evidence of immune cell trafficking into the CNS [10], and may occur independently of the formation of new lesions [11]. There is therefore a need to pursue supplemental MRI techniques, to gain a more accurate and comprehensive view of the pathogenesis of CNS inflammation. One strategy has been to employ iron oxide nanoparticles [12], particularly as a means of studying immune cell infiltration in the animal model of CNS inflammation, experimental autoimmune encephalomyelitis (EAE) [13]–[16]. However the application of paramagnetic nanoparticles is hampered by a number of limitations, including the lack of an a priori knowledge of the specific time of immune cell migration into the brain parenchyma.

Microscopic MRI (μMRI or MR histology) – defined as MRI with a spatial resolution <100 µm [17], [18] – is one means of amplifying image detail in order to observe even minor changes in brain pathology during the course of disease. MRI resolution depends on several factors including magnetic field strength, gradient strengths and digital resolution, but the main limiting factors are RF coil sensitivity and signal-to-noise ratio (SNR) [19]. Upon reducing voxel size to amplify spatial resolution, a loss in SNR is to be expected. This loss can be considerably compensated for by increasing signal averaging: this produces images with an impressive level of microscopic detail as shown in *ex vivo* fixed brain tissue samples [20]. However, increased signal averaging comes at a cost in scan time, and is hence not practical for studies with anesthetized animals. This along with the presence of movement artifacts makes it inherently difficult to achieve sufficient resolution to visualize brain pathology *in vivo*.

One approach to maximize effective spatial resolution is to increase signal sensitivity with cryogenically-cooled RF detection devices that boost SNR, the currency spent for image resolution and image quality [21]. Recently, RF coils made of superconducting material have been developed for animal micro-imaging, which reduce coil resistance and thermal noise and therefore increase SNR by up to a factor of up to 2.9 [22]. In essence such a system increases the field strength virtually by a factor of at least 2, according to MR principles, but without the disadvantages associated with higher field strengths, such as stronger susceptibility artifacts, B_0_ inhomogeneities and shorter wavelengths. The effectiveness of this approach has been demonstrated in the healthy mouse brain [23].

Realizing the capabilities of μMRI, we focused here on high spatial resolution imaging of mice brain during the course of EAE in an attempt to visualize inflammatory pathology *in vivo* prior to and during commencement of disease. The ultimate aim of this study was to distinguish cellular infiltrates in microscopic detail at an early phase of disease. In this first study applying cryogenic MR technology to EAE, we demonstrate that brain pathology can be detected even without the use of contrast agents and show excellent correspondence between μMRI findings and conventional histology.

## Results

### Early detection of EAE pathology with high resolution cryogenic imaging

**Figure 1:**
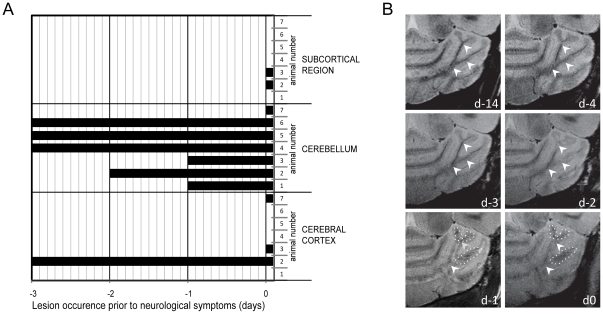
Anatomical distribution and evolution of lesions. EAE mice (n = 9) were immunized with PLP_139–151_ peptide (EAE induction) and scored for neurological symptoms on a daily basis. Micro MRI was performed daily starting from d5 post immunization. (A) Shown are seven mice (1–7) exhibiting lesions within the brain – specifically cerebral cortex, cerebellum and subcortical areas (thalamus and striatum) – prior to or during onset of disease. The lower axis indicates the time-points prior to commencement of neurological symptoms (d0). (B) T2W images showing the evolution of cerebellar lesions (arrowhead) from baseline (pre EAE induction) 14 days prior to disease manifestation (d-14) until disease onset (d0). Arrowheads depict enhancement of signal intensity in the white matter over time and dotted islands depict signal extinctions in the vicinity.

Using a cryogenically-cooled RF coil, we performed high spatial resolution brain imaging in EAE mice prior to and upon commencement of disease (**Figure S1**). The μMRI performed enabled us to detect lesions (as defined in Methods) already prior to clinical manifestation of disease. Using a T_2_ weighted (T2W) TurboRARE sequence that results in better contrast between grey and white matter boundaries, we observed lesions in multiple regions of the EAE mouse brains, particularly the cerebellum, cerebral cortex and subcortical regions (thalamus and striatum). **Figure 1A** shows the anatomical distribution of the detected lesions as well as the time point of their first detection (relative to the starting point of disease). Out of 9 immunized mice, all of which developed EAE, 7 mice exhibited lesions in the brain (**Figure S2**). The average EAE score for all 9 mice was 1.7±0.7 (± S.D.) and the average day of onset was 11.2±1.6 days (± S.D.) with a range of 9–14 days post immunization. All mice were scanned 5 days post immunization; thus between 4–9 days prior to disease onset. Notably, lesions could be detected as early as 3 days (d-3) prior to the appearance of disease manifestations (**Figure 1A**). No lesions were observed earlier than 3 days prior to clinical manifestations. Lesions in the cerebellum were the most common, occurring between 1 to 3 days prior to onset of disease (**Figure 1A**, **Figure 1B** and **Figure S3**). The T2W images shown in **Figure 1B** and **Figure S3** represent the structural changes occurring in the cerebellum in a mouse during the 4–5 days prior to disease onset (d-5 – d0) in comparison to baseline measurements (d-14) that were carried out prior to immunization. Hyperintense lesions in the white matter of the cerebellar *Arbor vitae* were already evident 2 days prior to neurological symptoms (d-2, upper and middle arrow head) and prominent on d-1 (along upper and lower arrowheads). Signal extinction that is indicative of cellular involvement became apparent on d-1 (dotted islands) but was more prominent upon onset of clinical symptoms (d0). To illustrate the potential for quantitative assessment of the lesions, we generated images in which we subtracted control baseline scans from scans showing brain pathology as changes in signal intensity (**Figure S4A**).

### Hypointense lesions on T2W images represent cellular infiltrates

**Figure 2:**
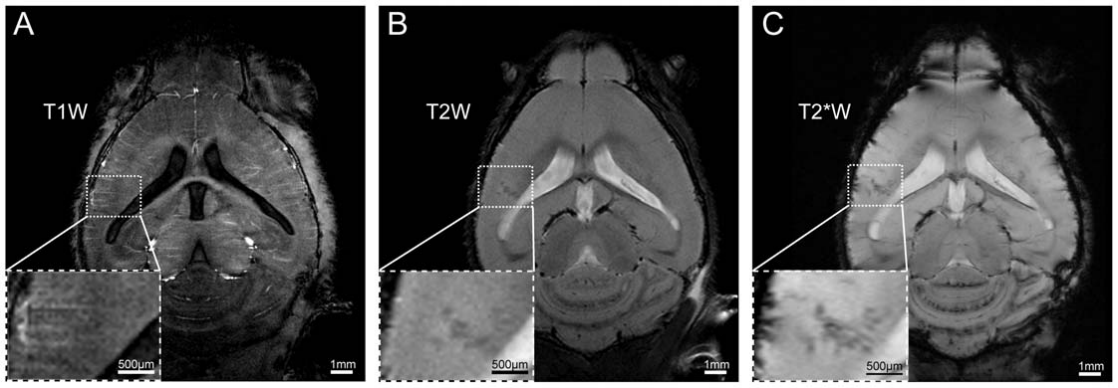
High resolution MRI reveals focal hypo-intense inflammation in the cerebral cortex of EAE mice. (A) T1W horizontal scan using an MDEFT sequence (TR/TE/TI 2600/3.9/950 ms, FOV 18×18 mm, Matrix 384×384, 2 averages) for 16 slices (400 µm slice-thickness). (B) T2W imaging using a TurboRARE sequence (TR/TE 3000/36 ms, FOV 18×18 mm, Matrix 384×384, RARE-factor 8) with same geometry and resolution as for T1W MDEFT. (C) T2*W imaging using a FLASH multislice sequence (TR/TE of 473/18 ms, FOV 18×18 mm, acquisition Matrix 512×256, reconstruction Matrix 512×512) with same slice thickness as in *A* and *B*.

**Figure 3:**
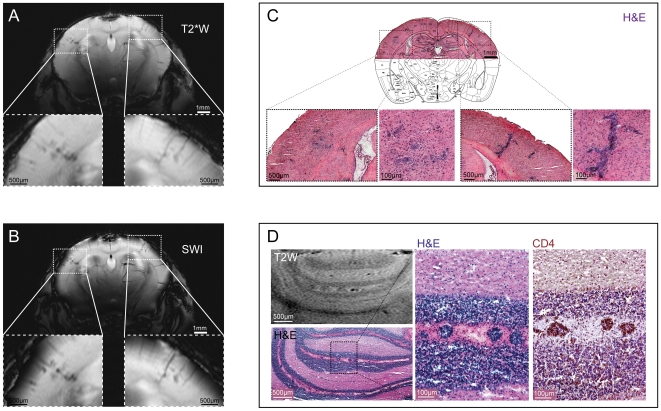
T2*W hypo-intense regions correspond to cellular infiltrates detected by histology. (A) Coronal T2*W imaging using the FLASH multislice sequence (22 slices with slice thickness of 500 µm). (B) Susceptibility weighted imaging (SWI) of T2*W scans using fully-automated post-processing by ParaVision 5.1 (Bruker, Ettlingen, Germany). (C) Cellular infiltrates in cerebral cortex; the overview of the H&E histology is overlaid with a coronal slice (plate 41) from Franklin K.B.J., Paxinos G: The Mouse Brain in Sterotaxic Coordinates. Academic Press; 2007 with kind permission from Elsevier. (D) Cellular infiltrates in cerebellar white matter lesions illustrated by CD4+ immunostaining and H&E staining.

Early during disease progression, even prior to the onset of clinical manifestations, we also observed lesions in the cortex. **Figure 2** illustrates a representative mouse at onset of clinically observable signs of disease. Although major anatomical structures including the corpus callosum, striatum, ventricular system and hippocampus could be clearly resolved with T_1_ weighted (T1W) scans (**Figure 2A**), no inflammatory pathology was observed with this method. T2W imaging, on the other hand, revealed focal hypo-intense lesions in multiple regions of the inflamed brains, mostly in the cerebellum (**Figure 1B**) but also in the somatosensory cortex (**Figure 2B**). T_2_* weighted (T2*W) imaging revealed the signal extinctions observed by T2W imaging as punctate lesions, with greater detail and in association with intracortical vessels (**Figure 2C** and **Figure 3A**). We observed venous irregularities within the cortex in 3 out of the 7 mice that exhibited lesions in the brain. The irregularities were observed one day prior to symptoms or upon initiation of symptoms. Processing of T2*W phase maps for susceptibility weighted imaging (SWI) [24] has been employed in MS lesions to reveal superior detail in small anatomical structures such as the venous vasculature and structural variations within gray and white matter [25]. SWI of the phase maps derived from T2*W imaging yielded excellent visualization of the small venous irregularities and enhanced contrast between normal tissue, focal inflammatory lesions and the microvasculature as highlighted in **Figure 3B**. To make a more objective assessment of these structural changes we subtracted the images showing pathology from images obtained at baseline, prior to EAE immunization (**Figure S4B**). Notably, the structural irregularities observed in T2W or T2*W images corresponded with areas of inflammatory pathology – both in cerebrum (**Figure 3C**) and cerebellum (**Figure 3D**) – as revealed by conventional hematoxylin and eosin (H&E) histology. H&E stains corroborate the μMRI data and conform to the expected pattern of pathology in the EAE model. The EAE model has been extensively characterized, and it is well established that the lesions are comprised of immune cells, especially CD4+ T cells. Indeed, the lesions that we could visualize with μMRI show the same appearance and localization characteristics of CD4+ T cells (**Figure 3D**). The clear correlation between the MRI data and the histology underscores the utility of microscopic MRI to reveal histologically relevant pathology *in vivo* [24], [25].

### Contrast enhanced lesions in close proximity to ventricular system

**Figure 4:**
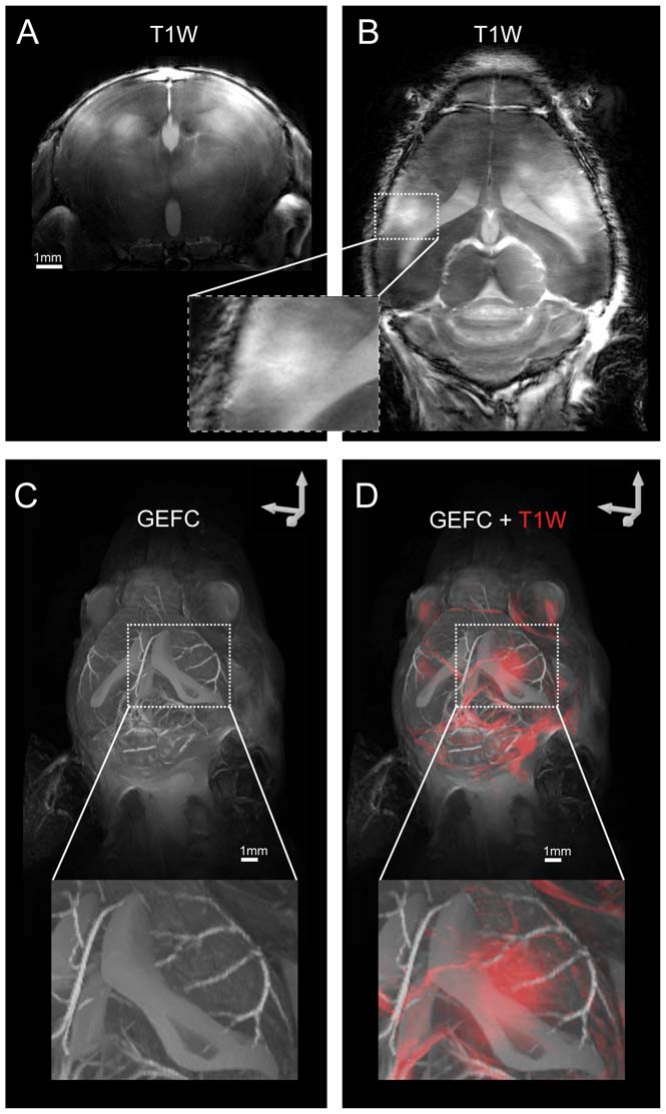
Three dimensional reconstruction of lesions in relation to the vascular and ventricular system. (A) Contrast-enhanced lesions (CEL) in coronal sections visualized by post Gd-DTPA contrast T1W MDEFT MRI (dotted line depicts horizontal slice in *B*). (B) CEL in horizontal sections imaged by post contrast T1W MDEFT (dotted line depicts coronal slice in *A*). (C) Maximum intensity projections (MIP) of images collected by 3D-GEFC showing the contrast-filled ventricular system and the cerebral vasculature (D) Overlay of a CEL 3D model (depicted in red) calculated from MIP of T1W MDEFT images on 3D ventricular and vascular system calculated from MIP of 3D-GEFC images.

Gadolinium contrast agents are frequently used in EAE studies, and Gd CEL have a typically diffuse appearance. T1W imaging post contrast (0.2 mmol/kg Gd-DTPA, Magnevist, Bayer-Schering Pharma AG, Berlin, Germany) revealed diffuse CEL (**Figure 4A** and **Figure 4B**) in all 7 mice exhibiting hyper- or hypo- intense lesions in the brain. These CEL, although more diffuse, corresponded in location to the T2W lesions shown by μMRI (**Figure 1** **,** **Figure 2** **,** **Figure 3**). This result corroborates the interpretation that the changes in signal intensities detected by T2W and T2*W microstructural MRI demonstrates the potential of microscopic MRI to reveal brain pathology with greater precision and detail than conventional contrast agents.

In EAE mice with lesions close to the ventricles, administration of Gd-DTPA enabled the visualization of the ventricular system (**Figure 4C**). The contrast-enriched ventricular system was visualized three-dimensionally together with the brain vasculature by performing a 29-minute 3D FLASH sequence with flow compensation (GEFC), typically employed for MR angiography, with an isotropic spatial resolution of 59 µm (**Figure 4C**). An overlay of the maximum intensity projections (MIP) of the 3D GEFC images with the MIP of post contrast T1W MDEFT CEL images shows the spatial relationship of the contrast-enhanced parenchyma (due to BBB disruption) with the ventricles and cerebral vasculature (**Figure 4D**). The visualization of the ventricular system together with the vasculature post contrast was only visible in EAE mice with periventricular lesions and was not visible in healthy non-immunized mice (**Figure S5**). While the precise mechanism still needs to be determined, these results suggest that an inflammation-induced disruption of the blood-cerebrospinal fluid barrier might lead to extravasation of contrast agent into the ventricular system.

## Discussion

Experimental autoimmune encephalomyelitis is an animal model widely employed to examine the basic biology of CNS inflammatory processes, and to evaluate the effectiveness of nascent therapeutic approaches for multiple sclerosis [26]–[31]. In particular, EAE studies focusing on the early stages of brain inflammation have revealed new insights on the mechanisms involved in the different waves of immune cell entry during evolution of disease [32].

To gain a comprehensive and longitudinal view of brain inflammation – particularly during the early stages of EAE – methods employing high resolution brain imaging would be advantageous. Several studies in EAE employing MRI have significantly contributed to our understanding of the pathology of brain inflammation [8], [16], [33], [34]. However, in longitudinal MRI studies (eg. to follow pathology during the course of EAE) one main limiting factor is a restriction in the number of animals that can be employed due to the length of each scanning procedure. To overcome this, methods that increase SNR will be beneficial to enable shorter scans. The gain in SNR achieved by cryogenically-cooled systems that reduce thermal noise [22] is a major advantage for studies with animal models for two main reasons: (i) it enables the acquisition of images with greater spatial resolution within reasonable scan times; and (ii) it facilitates the imaging of larger numbers of animals with standard spatial resolution but within shorter times. Depending on the design of the study it is possible to compensate between both strengths.

The advantages and feasibility of cryogenically cooled coils – particularly to image mouse brain anatomy – has already been demonstrated at 4.7T [35] and 9.4T [22], [23]. The scan time required using a conventional room-temperature coil to observe early pathological changes in the EAE brain (23 min for T2W imaging, 24 min for T2*W imaging) was at least three times longer than that of the currently employed cryogenically cooled coil (5 min for T2W imaging, 8 min for T2*W imaging) using the same magnetic field strength, considering the necessity to increase the number of excitations (NEX = 16 for RT coil compared with NEX = 1 for cryo coil) (**Figure S6**).

Handling of the currently available MRI cryogenic RF coils – which evolved from NMR-spectroscopy [21] – has significantly improved in recent years, and indeed is quite similar to any conventional RT RF-coil. One major drawback of the cryogenic probe used in this study is the transmission(TX)/reception(RX)-coil design, which leads to an inhomogeneous excitation field [22], [35]. The non-uniform excitation field needs to be taken into account when applying quantification techniques such as relaxometry or spectroscopy, as well as magnetisation transfer ratio (MTR). Due to the variation of the flip-angle throughout the sample, the reference gain has to be carefully adjusted for the volume of interest to minimize these changes. In this case a RX-only design with a conventional actively de-tuned TX volume resonator for a homogeneous excitation would be beneficial [36].

The effective anatomical resolution we employed in this study is almost an order of magnitude higher than that attainable in human brain imaging using clinical MRI scanners. Using this technology we observed structural changes in EAE brains – in regions including the cerebellum and cerebral cortex – prior to the onset of neurological symptoms. These detailed and highly resolved morphological changes were corroborated by subsequent histological examination, which showed clear evidence of infiltrated immune cells. The general trend in lesion distribution – with the cerebellum being the predominant site of damage in the brain – is in agreement with the ascending pattern of EAE. Disease progression in EAE shows a typical course of ascending paralysis, accompanied by inflammatory pathology that proceeds from the spinal cord initially affecting the tail and hind limbs, and subsequently precipitating complete paralysis. With this cryogenically-cooled system we were able to apply imaging protocols for high spatial resolution (as small as 35×35×400 µm), good grey matter–white matter distinction and high SNR with scan times not exceeding 15 min. The entire scanning protocol presented herein requires at most 60 min pre contrast and 60 min post contrast, and is therefore suitable for both longitudinal and cross-sectional *in vivo* studies, with acceptable sample sizes.

Importantly, we were able to identify the presence of inflammatory cells within the brain parenchyma without the necessity of Gd-DTPA contrast agents. In contrast to the typical diffuse lesions revealed by Gd enhancement, the lesions detected by μMRI were more focused and more clearly demarcated. This represents a clear advantage of the cryogenic coil over the previous technology, as the ability to detect focal lesions by μMRI offers the potential for more precise quantification of inflammation severity, leading to more quantitatively robust EAE studies. However contrast agents are still powerful tools since they provide clear evidence of BBB disruption, although leakage of contrast agent in the parenchyma does not give definitive evidence of parenchymal damage. In situations where structural changes observed in the brain with μMRI are not accompanied by contrast enhancement, it is reasonable to speculate that immune cells may have penetrated the vascular endothelium thereby entering the pervascular spaces, but have not yet breached the glia limitans and infiltrated into the brain parenchyma. Such perivascular-restricted lesions are widely seen at the histological level in EAE but have not yet been detected by MRI. The possibility of μMRI with the cryogenic coil to detect such lesions, which are by definition not detectable by Gd leakage into the parenchyma, offers the potential for future investigations to observe pathological processes that would otherwise be invisible, and underscores the importance of this technology for MS research.

Another distinct advantage of the cryogenic RF coil for MS research is the possibility to include magnetic resonance angiography in the scanning protocol, in a quick and efficient manner, to monitor changes in the vasculature over the course of disease. Indeed, the present study represents the first application of MR angiography in the mouse EAE model. The observation of leakage of contrast agent into the ventricular system early in disease is interesting, in light of recent reports highlighting the importance of the choroid plexus [30] and the involvement of the circumventricular organs in EAE [37].

In summary, the visualization of cellular infiltrates by *in vivo* μMRI provides an opportunity to follow neuroinflammatory processes throughout disease progression. Thus in parallel to conventional histological examination and in combination with contrast agents, μMRI will be invaluable for longitudinal studies investigating immune cell infiltration during brain inflammation and evaluation of novel therapeutics. Future directions for μMRI studies in EAE will involve the application of techniques – such as T2 mapping and MTR – that provide valuable information on water/lipid content and myelin integrity during lesion development. These techniques have already been successfully employed in a model of oligodendrogliopathy [38], [39] and will complement MR methods typically employed in EAE studies since they provide further knowledge regarding the neurodegenerative component of the disease.

## Materials and Methods

### Ethics Statement

Animal experiments were carried out in accordance with the guidelines provided and approved by the Animal Welfare Department of the *LAGeSo* State Office of Health and Social Affairs Berlin (Permit G0172/10).

### Active EAE

To actively induce EAE, 6–8 week old female SJL/J mice (Janvier, France) were immunized subcutaneously with 250 µg PLP_139–151_ purity >95% (Pepceuticals Ltd., UK) together with Complete Freund's Adjuvant and heat-killed Mycobacterium tuberculosis (H37Ra, Difco). Bordetella pertussis toxin (250 ng; List Biological Laboratories, US) was administered intraperitoneally at days 0 and 2. Mice were assigned a clinical score daily: 0, no disease; 1, tail weakness; 2, paraparesis; 3, paraplegia; 4, paraplegia with forelimb weakness; 5, moribund or dead animals.

### In vivo MRI

Shortly before and during the MR session, mice were anesthetized using a mixture of isoflurane as inhalation narcosis (0.5–1.5%), pressurized air and oxygen. All MRI-scans were performed on a Bruker Biospec 9.4T USR94/20 (Bruker, Ettlingen, Germany). Mice were imaged using a Transceiver Mouse Brain CryoProbe (Z106543, 400 MHz, Bruker BioSpin MRI, Ettlingen, Germany). The CryoProbe is a half-cylindrical-shaped 2-channel transmit/receive quadrature-driven surface coil, for full mouse-brain coverage with a maximum field of view of c. 30×20×20 mm. The temperature of the mice was regulated at 37°C. The breathing rate and temperature was monitored by a remote monitoring system (Model 1025, SA Instruments Inc., Syracuse, New York, USA). Images were acquired using T_1_-weighted (T1W), T_2_-weighted (T2W) and T_2_*-weighted (T2*W) techniques. Slice positioning was kept fixed through longitudinal brain examination: axial slices were positioned parallel to the base of the brain, coronal slices were positioned perpendicular to axial slices and covering the brain from the olfactory bulb/frontal lobe fissure to the cervical spinal cord.

### MRI sequences

Pre and post contrast T1W imaging was done with a Modified Driven-Equilibrium Fourier Transformation sequence (MDEFT) (3D MDEFT: TR/TE/TI: 3000/3.9/950 ms, FA 20°, matrix 384×384). Horizontal sections of the entire mice brain were performed in 11 min at a spatial resolution of (47×47×400) µm^3^. Coronal sections were performed in 15 min at a spatial resolution of (47×47×500) µm^3^. As T_1_-enhancing contrast agent gadolinium diethylenetriamine penta-acetate (Gd-DTPA, Magnevist, Bayer-Schering Pharma AG, Berlin, Germany) was administered intravenously at a concentration of 0.2 mmol/kg. T2W scans (2D TurboRARE: TR/TE: 3000/43 ms, matrix 384×384) were performed in 5 min with geometry and spatial resolution identical to that of T1W imaging. To image brain tissue in association with the microvasculature we applied a T2*W multislice fast low angle shot (2D FLASH: TR/TE: 473/18 ms, FA 40°, matrix 512×512) sequence with an in plane resolution of (35×35) µm^2^. For horizontal scans we used a slice thickness of 400 µm and 16 slices for full brain coverage with a scan time of 8 min and for coronal scans we used 22 slices of 500 µm with a scan time of 11 min to image the whole mouse brain. For mice showing leakage of contrast agent, a 29 min 3D FLASH sequence with flow compensation (GEFC: TR/TE: 30/5.9 ms, matrix 512×256×256) was applied, for visualization of the vasculature and ventricular system 1 hr after contrast injection at an isotropic spatial resolution of 59 µm.

The following MRI sequences were used for the baseline scans, prior to immunization: TurboRARE, MDEFT, 2D FLASH and 3D GEFC. Five days post-immunization, the mice underwent daily horizontal and coronal T2W (TurboRARE) scans. If no lesions or clinical signs were observed, the mouse was returned to the home cage and scanned again the next day. If hypo- or hyper-intense lesions were detected, then 2D FLASH and MDEFT pre-contrast scans of that mouse were done immediately. The mouse was then injected i.v. with Gd-DPTA, and post-contrast MDEFT, TurboRARE and 3D GEFC scans were done (15, 45, and 55 minutes following contrast injection, respectively). If the mouse did not show clinical signs, regardless of the observation of lesions, it was returned to the home cage and scanned again the next day. If the mouse did show clinical signs, this was the pre-defined endpoint, the mouse was sacrificed on the same day, and the brain extracted for histological comparison. Given the inherent variability in the time of onset of EAE, we elected to use this design (**Figure S1**), customized for each individual animal, rather than use a pre-selected timetable.

### Image analysis

Images from longitudinal experiments in mouse EAE were analyzed by three individuals who were blinded towards disease activity of corresponding mice. Lesions on T2W images were defined as described for T2W lesions in MS patients [40]. Briefly, lesions had to be clearly visible, non-artifactual areas of change in the signal intensity (signal enhancement or extinction on the grey background) of T2W images compared to baseline T2W images (taken prior to EAE immunization). To generate susceptibility weighted images post processing of the phase maps derived from T2*W imaging for was performed by fully-automated reconstruction using ParaVision 5.1 (Bruker, Ettlingen, Germany).

### Histology

After terminal anesthesia, mice were transcardially perfused with 20 ml PBS, then with 20 ml zinc fixation solution (0.5% zinc acetate, 0.5% zinc chloride, 0.05% calcium acetate). Brains were then extracted and subsequently post-fixed in zinc solution for 3 d at room temperature. The tissues were then cryoprotected by incubation overnight at 4 degrees in 30% sucrose in PBS, then embeded in O.C.T. and frozen in methylbutane with dry ice. The tissues were cut into 12 um sections on a cryostat, and stained with hematoxylin and eosin according to standard procedures. For immunostaining, tissue sections were blocked with avidin, biotin, and normal goat serum, then incubated overnight at 4°C with rat anti-mouse CD4 antibody (Invitrogen). The sections were then incubated with biotinylated goat anti-rat IgG antibody (Vector Laboratories), then streptavidin-conjugated peroxidase, and visualized with Vector NovaRED Peroxidase substrate (Vector Laboratories) and counterstained with hematoxylin.

## Supporting Information

Figure S1 **Flow chart illustrating the design of the μMRI study in EAE mice.** Following a baseline scan, mice were immunized and scanned daily (horizontal and coronal T2W MRI) 5 d post-immunization. More intensive scans were performed and contrast (Gd-DPTA) was i.v. applied when lesions were detected with T2W MRI.
(TIF)

Figure S2 **EAE scores compared with day of lesion occurrence.** The neurological score of each mouse is plotted against the day of first lesion occurrence (d-1 denotes that lesions were first observed one day prior to onset of symptoms).
(TIF)

Figure S3 **Cerebellar changes during course of EAE.** T2W images using a TurboRARE sequence (TR/TE 3000/43 ms, FOV 18×18 mm, Matrix 512×512 (384×384 for d-14), RARE-factor 8) showing the evolution of cerebellar lesions from baseline (pre EAE induction) 14 days prior to disease manifestation (d-14) until disease onset (d0) including daily scans starting from day 5 (d-5) prior disease onset. Dotted islands depict signal changes in the white-matter of the cerebellum.
(TIF)

Figure S4 **Parenchymal and vascular changes following EAE induction.** (A) T2W images using a TurboRARE sequence TR/TE 3000/43 ms, FOV 18×18 mm, Matrix 512×512 (384×384 for baseline), RARE-factor 8). Top row: baseline; middle row: pre-symptomatic image revealing a hyperintense lesion in the cortex; bottom row: subtracted image. (B) SWI processed T2*W images using a FLASH multislice sequence (TR/TE of 473/18 ms, FOV 18×18 mm, acquisition Matrix 512×512). Top row: baseline; middle row: pre-symptomatic image showing vascular irregularities in the region of the T2 hyperintense lesion shown in (A); bottom row: subtracted image.
(TIF)

Figure S5 **Maximum intensity projections (MIP) pre and post contrast administration in a healthy non-immunized mouse.** (A) Pre-contrast MIP of a 3D-GEFC sequence (TR/TE: 30/5.9 ms, matrix 512×256×256). (B) Post-contrast MIP of the same mouse.
(TIF)

Figure S6 **Differences in quality and scan duration between images showing cortical hypointense lesions in EAE brains using either a cryogenically cooled coil (A, C) or a room temperature (RT) 18 mm mouse-head birdcage coil (B, D).** (A) T2W images using a TurboRARE sequence with cryogenic coil (TR/TE 3000/36 ms, FOV 18×18 mm, Matrix 384×384) (B) T2W images using a TurboRARE sequence with RT coil (TR/TE 2000/30 ms, FOV 24×14 mm, Matrix 300×174) (C) T2*W images using a FLASH multislice sequence with cryo coil (TR/TE of 473/18 ms, FOV 18×18 mm, acquisition Matrix 512×512) (D) T2*W images using a FLASH multislice sequence with RT coil (TR/TE of 473/13 ms, FOV 24×14 mm, acquisition Matrix 330×192).
(TIF)

## References

[1] Trapp BD, Nave KA (2008). Multiple sclerosis: an immune or neurodegenerative disorder?. Annu Rev Neurosci.

[2] Stadelmann C (2011). Multiple sclerosis as a neurodegenerative disease: pathology, mechanisms and therapeutic implications.. Curr Opin Neurol.

[3] Charil A, Filippi M (2007). Inflammatory demyelination and neurodegeneration in early multiple sclerosis.. J Neurol Sci.

[4] Engelhardt B, Coisne C (2011). Fluids and barriers of the CNS establish immune privilege by confining immune surveillance to a two-walled castle moat surrounding the CNS castle.. Fluids Barriers CNS.

[5] Alvarez JI, Cayrol R, Prat A (2011). Disruption of central nervous system barriers in multiple sclerosis.. Biochim Biophys Acta.

[6] Hauser SL, Waubant E, Arnold DL, Vollmer T, Antel J (2008). B-cell depletion with rituximab in relapsing-remitting multiple sclerosis.. N Engl J Med.

[7] Paul F, Waiczies S, Wuerfel J, Bellmann-Strobl J, Dorr J (2008). Oral high-dose atorvastatin treatment in relapsing-remitting multiple sclerosis.. PLoS ONE.

[8] Wuerfel J, Tysiak E, Prozorovski T, Smyth M, Mueller S (2007). Mouse model mimics multiple sclerosis in the clinico-radiological paradox.. Eur J Neurosci.

[9] Levy H, Assaf Y, Frenkel D (2010). Characterization of brain lesions in a mouse model of progressive multiple sclerosis.. Exp Neurol.

[10] Vellinga MM, Oude Engberink RD, Seewann A, Pouwels PJ, Wattjes MP (2008). Pluriformity of inflammation in multiple sclerosis shown by ultra-small iron oxide particle enhancement.. Brain.

[11] Wuerfel J, Bellmann-Strobl J, Brunecker P, Aktas O, McFarland H (2004). Changes in cerebral perfusion precede plaque formation in multiple sclerosis: a longitudinal perfusion MRI study.. Brain.

[12] Weissleder R, Cheng HC, Bogdanova A, Bogdanov A (1997). Magnetically labeled cells can be detected by MR imaging.. J Magn Reson Imaging.

[13] Dousset V, Gomez C, Petry KG, Delalande C, Caille JM (1999). Dose and scanning delay using USPIO for central nervous system macrophage imaging.. MAGMA.

[14] Floris S, Blezer EL, Schreibelt G, Dopp E, van der Pol SM (2004). Blood-brain barrier permeability and monocyte infiltration in experimental allergic encephalomyelitis: a quantitative MRI study.. Brain.

[15] Oude Engberink RD, Blezer EL, Dijkstra CD, van der Pol SM, van der TA (2010). Dynamics and fate of USPIO in the central nervous system in experimental autoimmune encephalomyelitis.. NMR Biomed.

[16] Tysiak E, Asbach P, Aktas O, Waiczies H, Smyth M (2009). Beyond blood brain barrier breakdown - in vivo detection of occult neuroinflammatory foci by magnetic nanoparticles in high field MRI.. J Neuroinflammation.

[17] Aguayo JB, Blackband SJ, Schoeniger J, Mattingly MA, Hintermann M (1986). Nuclear magnetic resonance imaging of a single cell.. Nature.

[18] Benveniste H, Blackband S (2002). MR microscopy and high resolution small animal MRI: applications in neuroscience research.. Prog Neurobiol.

[19] Hoult DI, Richards RE (1976). The signal-to-noise ratio of the nuclear magnetic resonance experiment.. J Magn Reson (1969).

[20] Cleary JO, Wiseman FK, Norris FC, Price AN, Choy M (2011). Structural correlates of active-staining following magnetic resonance microscopy in the mouse brain.. Neuroimage.

[21] Kovacs H, Moskau D, Spraul M (2005). Cryogenically cooled probes - a leap in NMR technology.. Prog Nucl Magn Reson Spectrosc.

[22] Nouls JC, Izenson MG, Greeley HP, Johnson GA (2008). Design of a superconducting volume coil for magnetic resonance microscopy of the mouse brain.. J Magn Reson.

[23] Baltes C, Radzwill N, Bosshard S, Marek D, Rudin M (2009). Micro MRI of the mouse brain using a novel 400 MHz cryogenic quadrature RF probe.. NMR Biomed.

[24] Haacke EM, Xu Y, Cheng YC, Reichenbach JR (2004). Susceptibility weighted imaging (SWI).. Magn Reson Med.

[25] Haacke EM, Makki M, Ge Y, Maheshwari M, Sehgal V (2009). Characterizing iron deposition in multiple sclerosis lesions using susceptibility weighted imaging.. J Magn Reson Imaging.

[26] Steinman L, Zamvil SS (2006). How to successfully apply animal studies in experimental allergic encephalomyelitis to research on multiple sclerosis.. Ann Neurol.

[27] Ben Nun A, Wekerle H, Cohen IR (1981). Vaccination against autoimmune encephalomyelitis with T-lymphocyte line cells reactive against myelin basic protein.. Nature.

[28] Hickey WF, Kimura H (1988). Perivascular microglial cells of the CNS are bone marrow-derived and present antigen in vivo.. Science.

[29] Engelhardt B, Wolburg-Buchholz K, Wolburg H (2001). Involvement of the choroid plexus in central nervous system inflammation.. Microsc Res Tech.

[30] Reboldi A, Coisne C, Baumjohann D, Benvenuto F, Bottinelli D (2009). C-C chemokine receptor 6-regulated entry of TH-17 cells into the CNS through the choroid plexus is required for the initiation of EAE.. Nat Immunol.

[31] Bartholomaus I, Kawakami N, Odoardi F, Schlager C, Miljkovic D (2009). Effector T cell interactions with meningeal vascular structures in nascent autoimmune CNS lesions.. Nature.

[32] Ransohoff RM (2009). Immunology: In the beginning.. Nature.

[33] Budde MD, Kim JH, Liang HF, Russell JH, Cross AH (2008). Axonal injury detected by in vivo diffusion tensor imaging correlates with neurological disability in a mouse model of multiple sclerosis.. NMR Biomed.

[34] Nessler S, Boretius S, Stadelmann C, Bittner A, Merkler D (2007). Early MRI changes in a mouse model of multiple sclerosis are predictive of severe inflammatory tissue damage.. Brain.

[35] Ratering D, Baltes C, Nordmeyer-Massner J, Marek D, Rudin M (2008). Performance of a 200-MHz cryogenic RF probe designed for MRI and MRS of the murine brain.. Magn Reson Med.

[36] Wright AC, Song HK, Wehrli FW (2000). In vivo MR micro imaging with conventional radiofrequency coils cooled to 77 degrees K.. Magn Reson Med.

[37] Wuerfel E, Infante-Duarte C, Glumm R, Wuerfel JT (2010). Gadofluorine M-enhanced MRI shows involvement of circumventricular organs in neuroinflammation.. J Neuroinflammation.

[38] Mueggler T, Pohl H, Baltes C, Riethmacher D, Suter U (2011). MRI signature in a novel mouse model of genetically induced adult oligodendrocyte cell death.. Neuroimage.

[39] Pohl HB, Porcheri C, Mueggler T, Bachmann LC, Martino G (2011). Genetically induced adult oligodendrocyte cell death is associated with poor myelin clearance, reduced remyelination, and axonal damage.. J Neurosci.

[40] Tan IL, van Schijndel RA, Fazekas F, Filippi M, Freitag P (2002). Image registration and subtraction to detect active T(2) lesions in MS: an interobserver study.. J Neurol.

