# Supplementary figures and images for: Identification of Cellular Infiltrates during Early Stages of Brain Inflammation with Magnetic Resonance Microscopy
Source: PLoS One. 2012 Mar 12;7(3):e32796. doi: 10.1371/journal.pone.0032796 (PMC3299701; doi:10.1371/journal.pone.0032796)

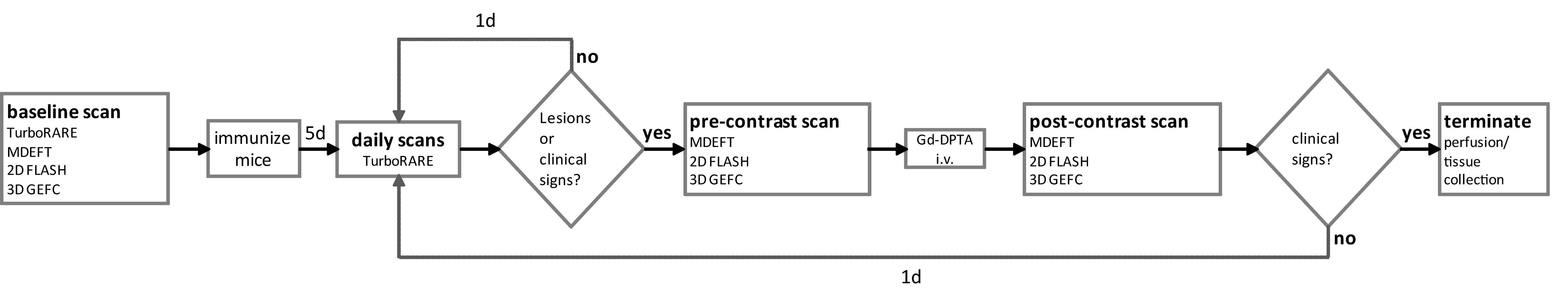

Supplement: Figure S1 — Flow chart illustrating the design of the μMRI study in EAE mice. Following a baseline scan, mice were immunized and scanned daily (horizontal and coronal T2W MRI) 5 d post-immunization. More intensive scans were performed and contrast (Gd-DPTA) was i.v. applied when lesions were detected with T2W MRI. (TIF) [file pone.0032796.s001.tif]

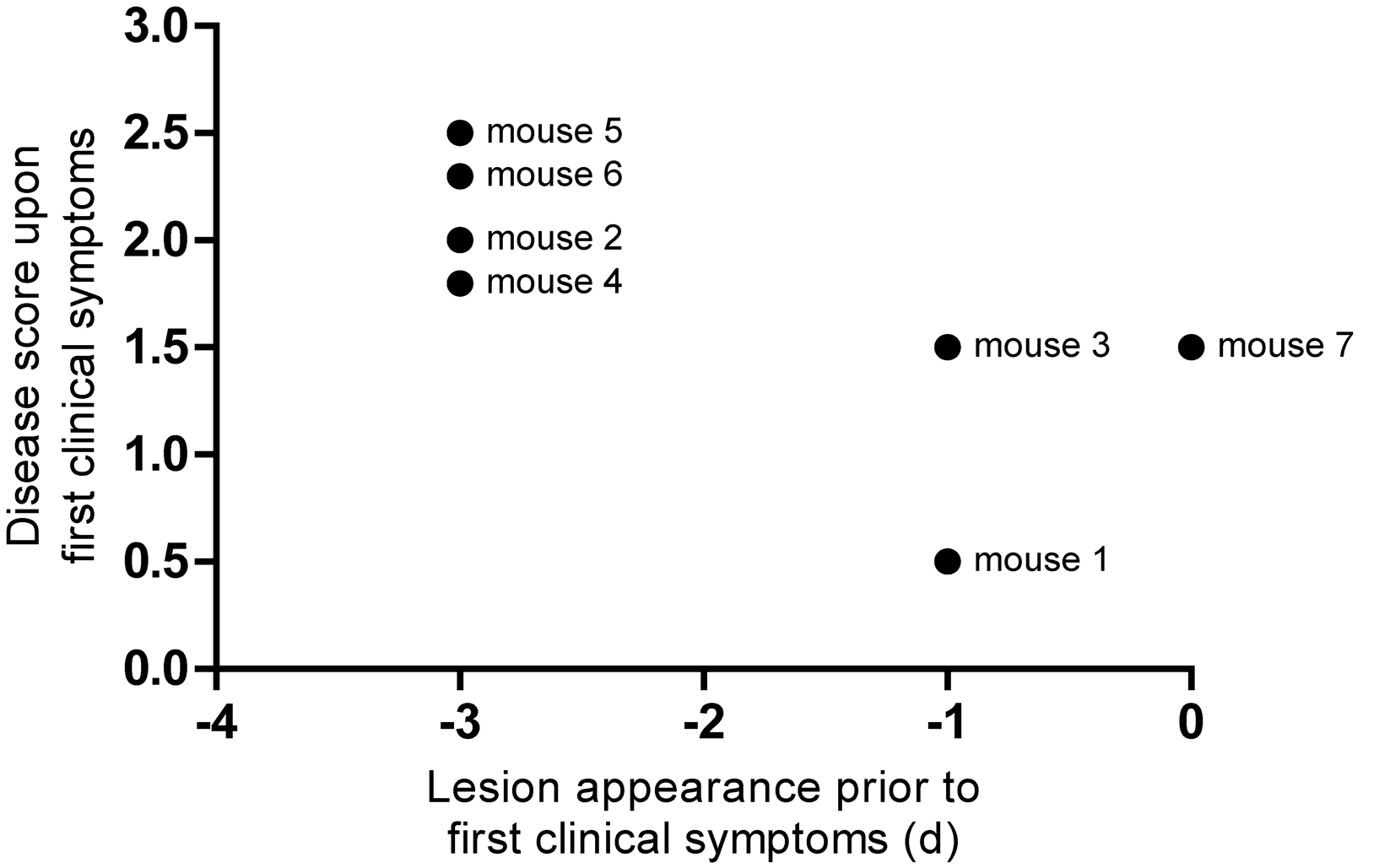

Supplement: Figure S2 — EAE scores compared with day of lesion occurrence. The neurological score of each mouse is plotted against the day of first lesion occurrence (d-1 denotes that lesions were first observed one day prior to onset of symptoms). (TIF) [file pone.0032796.s002.tif]

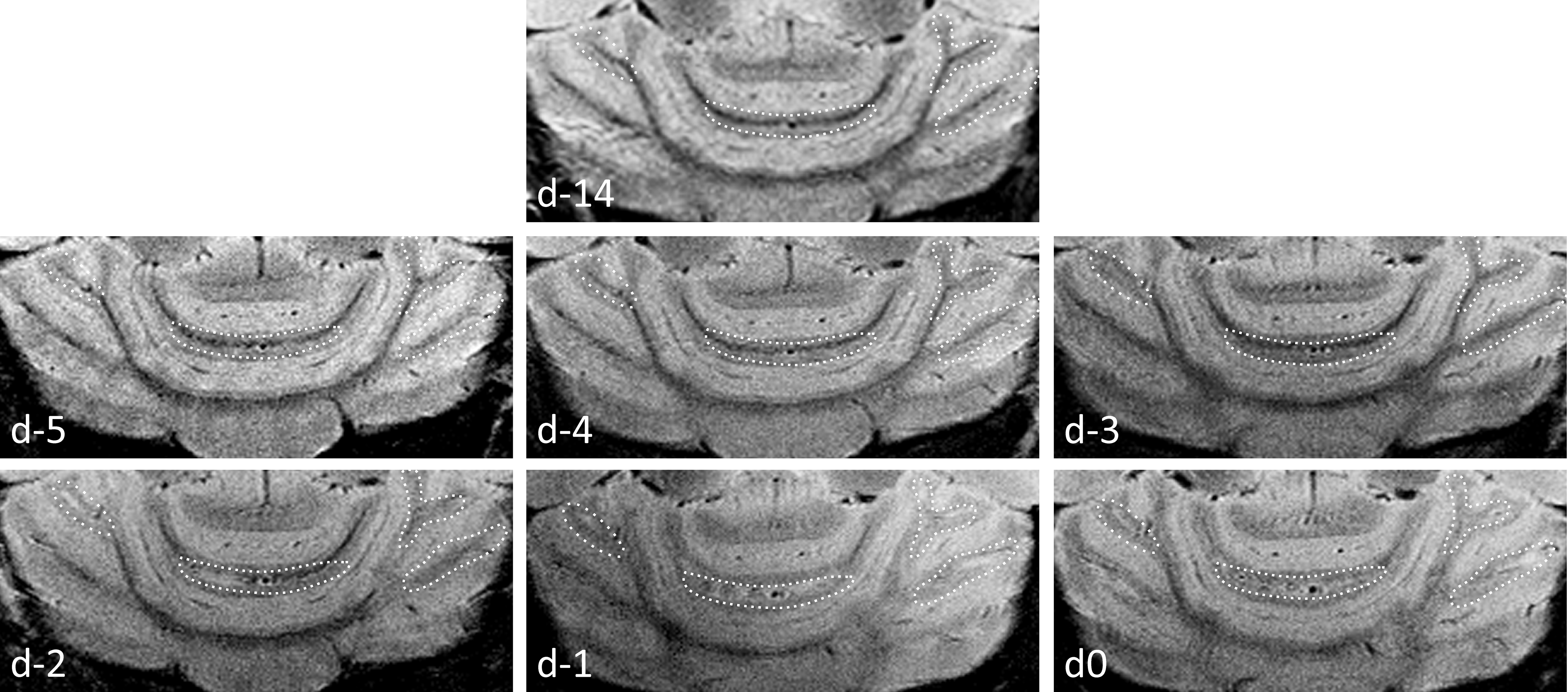

Supplement: Figure S3 — Cerebellar changes during course of EAE. T2W images using a TurboRARE sequence (TR/TE 3000/43 ms, FOV 18×18 mm, Matrix 512×512 (384×384 for d-14), RARE-factor 8) showing the evolution of cerebellar lesions from baseline (pre EAE induction) 14 days prior to disease manifestation (d-14) until disease onset (d0) including daily scans starting from day 5 (d-5) prior disease onset. Dotted islands depict signal changes in the white-matter of the cerebellum. (TIF) [file pone.0032796.s003.tif]

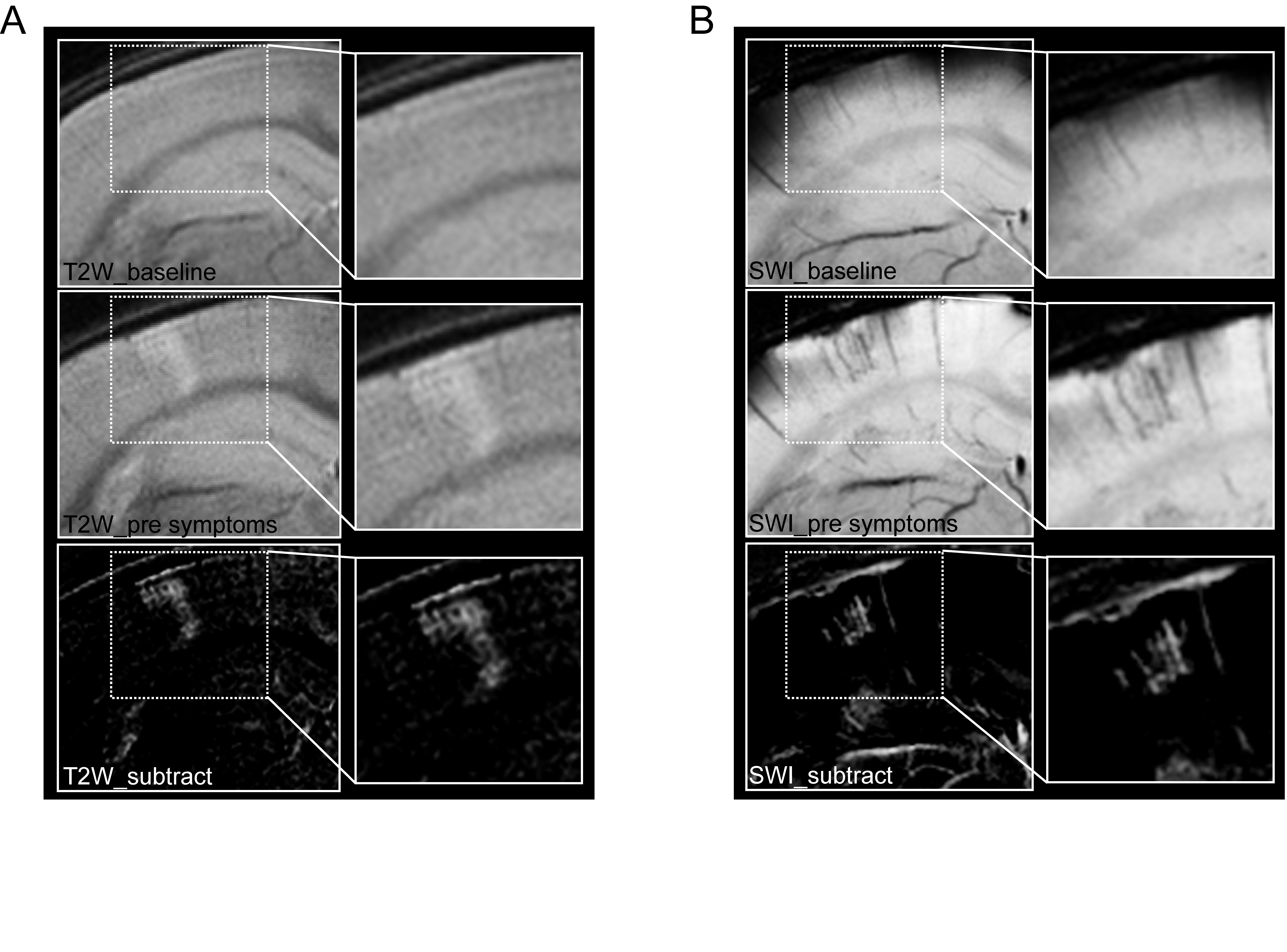

Supplement: Figure S4 — Parenchymal and vascular changes following EAE induction. (A) T2W images using a TurboRARE sequence TR/TE 3000/43 ms, FOV 18×18 mm, Matrix 512×512 (384×384 for baseline), RARE-factor 8). Top row: baseline; middle row: pre-symptomatic image revealing a hyperintense lesion in the cortex; bottom row: subtracted image. (B) SWI processed T2*W images using a FLASH multislice sequence (TR/TE of 473/18 ms, FOV 18×18 mm, acquisition Matrix 512×512). Top row: baseline; middle row: pre-symptomatic image showing vascular irregularities in the region of the T2 hyperintense lesion shown in (A); bottom row: subtracted image. (TIF) [file pone.0032796.s004.tif]

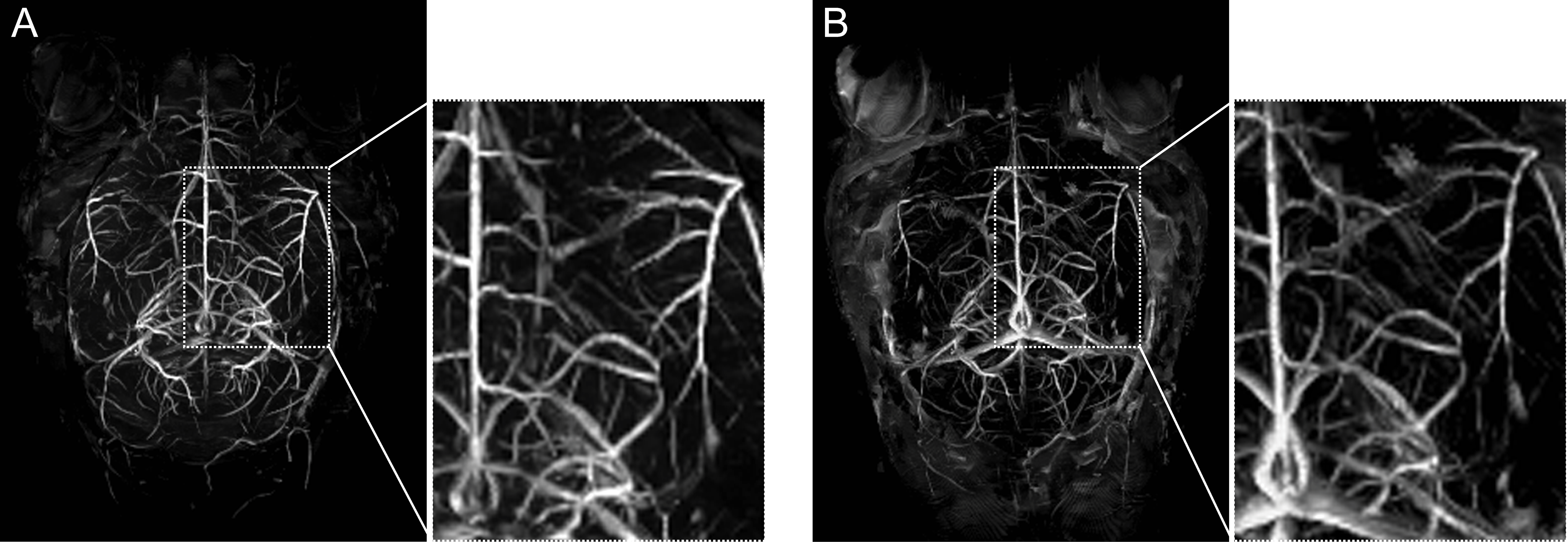

Supplement: Figure S5 — Maximum intensity projections (MIP) pre and post contrast administration in a healthy non-immunized mouse. (A) Pre-contrast MIP of a 3D-GEFC sequence (TR/TE: 30/5.9 ms, matrix 512×256×256). (B) Post-contrast MIP of the same mouse. (TIF) [file pone.0032796.s005.tif]

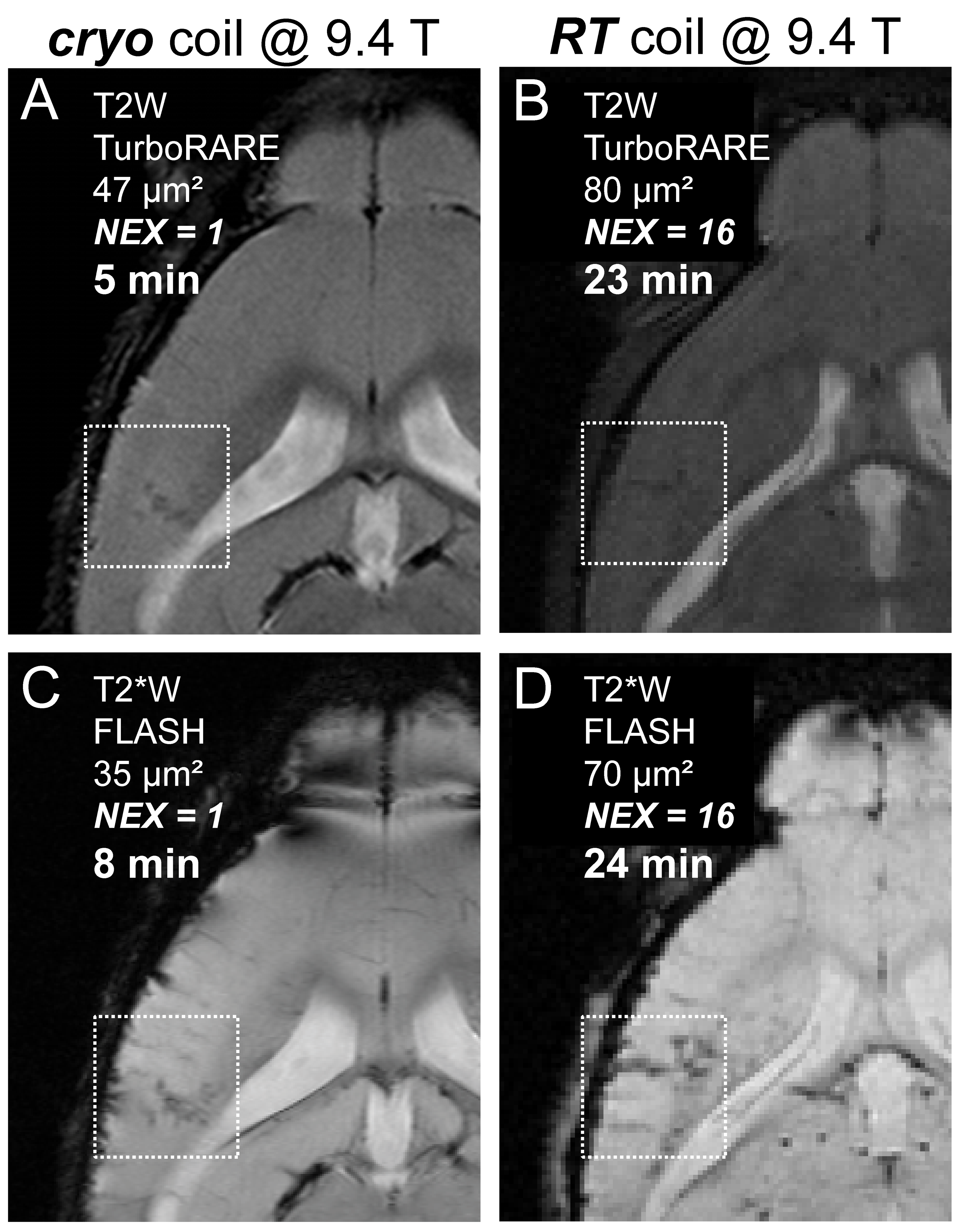

Supplement: Figure S6 — Differences in quality and scan duration between images showing cortical hypointense lesions in EAE brains using either a cryogenically cooled coil (A, C) or a room temperature (RT) 18 mm mouse-head birdcage coil (B, D). (A) T2W images using a TurboRARE sequence with cryogenic coil (TR/TE 3000/36 ms, FOV 18×18 mm, Matrix 384×384) (B) T2W images using a TurboRARE sequence with RT coil (TR/TE 2000/30 ms, FOV 24×14 mm, Matrix 300×174) (C) T2*W images using a FLASH multislice sequence with cryo coil (TR/TE of 473/18 ms, FOV 18×18 mm, acquisition Matrix 512×512) (D) T2*W images using a FLASH multislice sequence with RT coil (TR/TE of 473/13 ms, FOV 24×14 mm, acquisition Matrix 330×192). (TIF) [file pone.0032796.s006.tif]
